# Dl-3-n-Butylphthalide (NBP) Mitigates Muscular Injury Induced by Limb Ischemia/Reperfusion in Mice through the HMGB1/TLR4/NF-*κ*B Pathway

**DOI:** 10.1155/2022/5556067

**Published:** 2022-09-26

**Authors:** Huanhuan Sun, Jueqiong Wang, Wei Bi, Feng Zhang, Kui Chi, Long Shi, Meng Li, Jinwen Zhang, Yanrong Zhang, Xiang Gao

**Affiliations:** ^1^Department of Vascular Surgery, The Second Hospital of Hebei Medical University, Shijiazhuang 050000, Hebei, China; ^2^Department of Neurology, Neurological Laboratory of Hebei Province, The Second Hospital of Hebei Medical University, Shijiazhuang 050000, Hebei, China; ^3^Department of Vascular Surgery, The Third Hospital of Hebei Medical University, Shijiazhuang 050000, Hebei, China

## Abstract

**Objective:**

Limb ischemia/reperfusion (I/R) injury is a clinical syndrome associated with severe damages to skeletal muscles and other fatal outcomes. Oxidative stress and inflammatory response play vital roles in the development of limb I/R injury. Existing evidence further indicates that Dl-3-n-butylphthalide (NBP) has anti-inflammatory and antioxidative properties. However, whether NBP can protect skeletal muscles from limb I/R injury and the mechanism in mediating the action of NBP treatment still remain to be investigated, which are the focuses of the current study.

**Methods:**

The model of limb I/R injury was established and H&E staining was adopted to assess the pathological changes in skeletal muscles following limb I/R injury. Additionally, the W/D ratio of muscle tissue was also measured. ELISA and biochemical tests were carried out to measure the levels of inflammatory cytokines and oxidative stress in mouse models of limb I/R injury. Moreover, the levels of the HMGB1/TLR4/NF-*κ*B pathway-related proteins were also determined using immunohistochemistry and immunoblotting.

**Results:**

It was established that NBP treatment alleviated I/R-induced pathological changes in muscular tissue of mice, accompanied by lower W/D ratio of skeletal muscular tissue. Meanwhile, the limb I/R-induced inflammation and oxidative stress in skeletal muscles of mice were also inhibited by NBP. Mechanistic study indicated that the alleviatory effect of NBP was ascribed to inactivation of the HMGB1/TLR4/NF-*κ*B pathway.

**Conclusions:**

Our findings highlighted the potential of NBP as a novel strategy for limb I/R-driven muscle tissue damages by suppressing inflammatory response and oxidative stress *via* the HMGB1/TLR4/NF-*κ*B pathway.

## 1. Introduction

Limb ischemia/reperfusion (I/R) injury is classified as a clinical syndrome with fatal outcomes for humans. Significant advancements in the understanding of limb I/R injury have shown that arterial embolism, trauma, blood clots, abdominal compartment syndrome, prolonged tourniquet application, and limb or flap reattachment serve as the primary causes of limb I/R injury [1]. Skeletal muscles are particularly susceptible to limb I/R injury; even moderate I/R injuries can precipitate permanent damage and necrosis of skeletal muscles, which could ultimately exert detrimental effects on limb functionality. Unfortunately, some patients must undergo amputation to save their lives in severe cases. Further adding to the plight, multiple organ dysfunction syndrome(MODS) is a common occurrence in patients with critical limb I/R injury, which represents a fatal condition for patients [2].

Pathologically, a plethora of mechanisms are known to be associated with skeletal muscle damage caused by limb I/R injury. However, inflammatory response and oxidative stress play critical roles in advancing skeletal muscle I/R injury [3, 4]. Organs involved in I/R injury present with enhanced production of reactive oxygen species (ROS), whereas the overproduction of ROS is previously associated with diminished expression of antioxidative proteins related to stress, which further exacerbates I/R injury [4]. Typically, malondial-dehyde (MDA) and super-oxide dismutase (SOD) are commonly utilized as indicators of oxidative stress [5]. Moreover, injuries induced by ROS are known to release proinflammatory cytokines [6].

In the course of distinct cellular processes, including cell damage, cell death, and cytokine stimulation, an ubiquitous DNA-binding nuclear protein, known as high mobility group protein B1 (HMGB1), can be secreted into an extracellular region as a factor with effective proinflammatory features [7]. It should be noted that HMGB1 has been previously shown to enhance inflammatory responses in microvascular injury of endothelial cells *via* the release of proinflammatory cytokines in the blood of septic patients [8]. Moreover, there is evidence to suggest that HMGB1 proteins carry out their biological functions by binding with cell-surface receptors, specifically known as toll-like receptors (TLRs) [7]. TLRs are classified under pattern recognition receptors (PRRs), which could possess the ability to activate innate and adaptive immune responses [9]. Currently, approximately thirteen members of TLRs have been discovered and identified in mammals. Out of these, TLR4 is the first to be recognized for its ability to bind with lipopolysaccharide and produce proinflammatory cytokines; moreover, among all members of the TLRs, TLR4 has also been the most extensively characterized and widely expressed receptor [10]. Accumulating evidence has shown that TLR4 exhibits a significant role in both innate and adaptive immune responses. In addition, TLR4 is previously documented to mediate organ injuries in various I/R models [11]. Strikingly, the efforts of Oklu *et al.* have demonstrated that TLR4 exerts vital influence in the pathogenesis of limb I/R injury [12]. TLR4 triggered by limb I/R injury is further established to activate nuclear factor (NF)-*κ*B *via* myeloid differentiation factor 88 (Myd88)-dependent pathway, resulting in release of proinflammatory cytokines, eventually leading to aggravation of tissue damage [13].

Dl-3-n-butylphthalide (NBP) is obtained from *Apium graveolens* Linn seeds, commonly known as Chinese celery, are widely adopted in clinical settings to treat ischemic stroke. NBP has numerous therapeutic benefits, which include antioxidant [14], antiapoptotic [15], and anti-inflammatory properties [16]. Additionally, prior evidence indicates that the administration of NBP can alleviate cerebral I/R-induced brain injury and spinal cord injury through TLR4/NF-*κ*B inhibition [17]. However, whether NBP has the ability to safeguard skeletal muscle from limb I/R injury, and the potential underlying mechanism that mediate the action of NBP treatment remain to be studied. Accordingly, the current study is aimed to elucidate protective effects of NBP on the skeletal muscle against limb I/R injury and to clarify the underlying signaling pathway that mediates the beneficial effects of NBP.

## 2. Materials and Methods

### 2.1. Experimental Animals

Firstly, male C67/BL6 mice [23–25 g, 8–10 weeks; SPF (Beijing) Biotechnology Co., Ltd., Beijing, China] were reared under SPF conditions, with ad libitum access to food and water. Animal experimentation was carried out in line with Guide for the Care and Use of Laboratory Animals published by the National Institutes of Health following approval by the Ethics Committee for Animal Use of Hebei Medical University.

### 2.2. Mouse Model Generation of Femoral Artery I/R Injury

The femoral artery and vein were exposed, and the supply of blood was blocked and a band was fitted around the left thigh to induce ischemia for 1.5 h. Afterward, the clamp together with the band was removed for reperfusion induction for 72 h prior to sampling.

### 2.3. Experimental Groups and Drug Treatment

Following femoral artery I/R modeling, the mice were assigned into sham, I/R, and I/R + NBP (40 mg/kg; Bide Pharmatech Ltd., Shanghai, China) groups. The femoral artery and vein of the mice in the sham group were blocked for 1.5 h, and the mice were intraperitoneally injected with saline prior to reperfusion on the day of the surgery, followed by intraperitoneal saline administration once on a daily basis for two additional days prior to sampling. Meanwhile, the femoral artery and vein of the mice in the I/R group were blocked for 1.5 h, following which the mice were intraperitoneally injected with saline prior to reperfusion on the operation day, and the mice received intraperitoneal administration with saline once per day for two days before sampling. In the I/R + NBP group, the femoral artery and vein of mice were blocked for 1.5 h. Afterward, the mice received intraperitoneal administration with saline via injection containing 40 mg/kg NBP before reperfusion on the operation day and the drug treatment was given once per day for two days before sampling. Afterward, all the mice were euthanized by exsanguination, followed by tissue collection.

### 2.4. Histology and Immunohistochemistry

Following 3 days of administration, pretibial muscle tissues were removed and paraffin-embedded. Subsequently, the sections (5 *μ*m) were subjected to staining with H&E to assess the general histology and inflammation as previously described [4, 6].

Immunohistochemistry was carried out to measure HMGB1 and TLR4 expression patterns in pretibial muscle tissue sections (5 *μ*m). Initially, the sections were deparaffinized and rehydrated prior to treatment with 3% (v/v) H_2_O_2_ in methanol, followed by blocking with BSA. The sections were probed with antibodies to HMGB1 (ab18256; 1 : 300; Abcam) and TLR4 (ab217274; 1 : 200; Abcam). Once the sections were washed, bound antibodies were recognized with biotin labeled secondary antibodies and an ABC kit. A light microscope was utilized to visualize the staining.

### 2.5. Wet/Dry (W/D) Weight Ratio of Muscle Tissue

Pretibial muscle was excised from the left hind limb, it was instantly weighed and recorded as wet weight. Subsequently, the muscle was dehydrated and then weighed again as dry weight. W/D ratio = wet weight/dry weight [4, 6].

### 2.6. Enzyme-Linked Immunosorbent Assay (ELISA)

Using ELISA kits (ELK1271, ELK1395, ELK1157; acquired from ELK Biotechnology, Wuhan, China), levels of TNF-*α*, IL-1*β*, and IL-6 in the homogenates of muscular tissues of each group of mice were determined.

### 2.7. Measurement of LDH, CK-MB, MDA, and SOD

The muscular tissues (0.1 g) were weighed and immersed in 900 *μ*l of normal saline via ultrasonic trituration on ice to obtain muscular homogenates. MDA production was evaluated with MDA detection kits (A003-1, Nanjing Jiancheng Biotechnology Institute, Nanjing, China). Additionally, activity of SOD (A001-3, Nanjing Jiancheng Biotechnology Institute, Nanjing, China), LDH (A020-1, Nanjing Jiancheng Biotechnology Institute, Nanjing, China), and CK-MB (E006-1-1, Nanjing Jiancheng Biotechnology Institute, Nanjing, China) in muscular homogenates was all measured by commercial kits.

### 2.8. Immunoblotting

Muscular tissues were lysed, separated, and transferred to membranes. The membrane was probed with primary antibodies to HMGB1 (ab18256; 1 : 1000; Abcam, Cambridge, UK), TLR4 (ab217274; 1 : 1000; Abcam, Cambridge, UK), Myd88 (#4283; 1 : 1000; Cell Signaling Technology [CST], Beverly, USA), p-p65 (#3033; 1 : 500, CST, Beverly, USA), p65 (#8242; 1 : 1500, CST, Beverly, USA), and GAPDH (ab37168; 1 : 10000; Abcam, Cambridge, UK). The membrane was incubated with secondary antibody (1 : 10,000; ASPEN, Wuhan, China). Afterward, the Image J software was utilized for quantitative analysis of band intensities.

### 2.9. Statistical Analysis

SPSS 19.0 software was adopted for analysis of all results, which are presented as mean ± SD. One-way ANOVA and SNK-q test or the Dunnett's multiple comparison test was applied for multigroup comparison. *p* < 0.05 suggests statistical significance.

## 3. Results

### 3.1. NBP Alleviated I/R Injury-Driven Skeletal Muscle Damage

**Figure 1:**
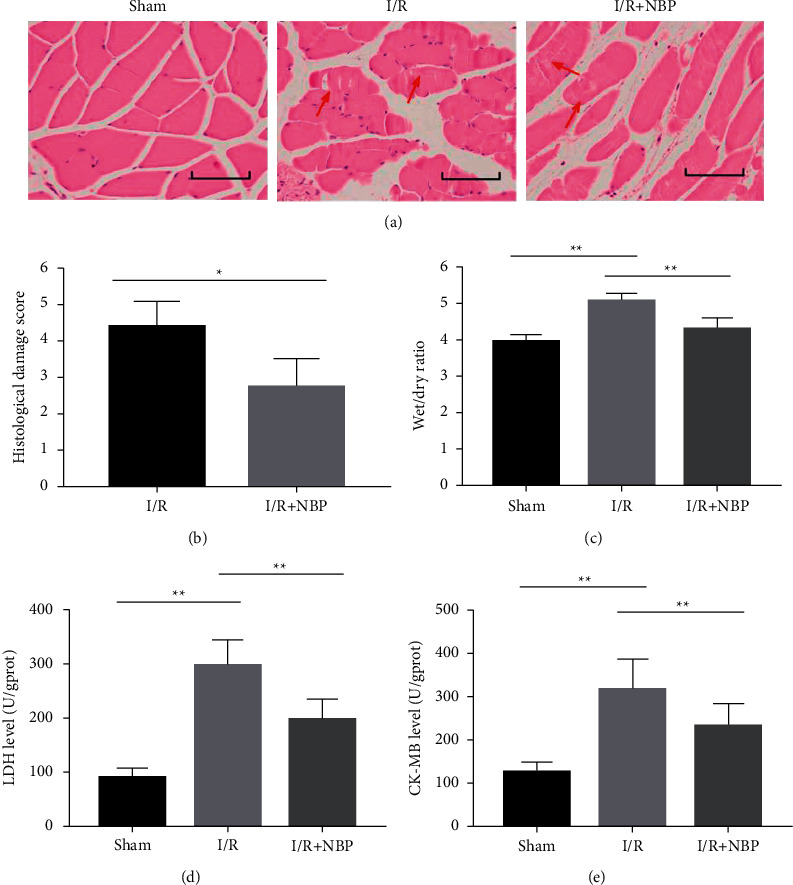
Effect of NBP on pathological change and wet weight/dry weight ratio of skeletal muscle in I/R-injured mice. (a) H&E staining was used to observe the degree of inflammation of muscular tissue in each group of mice (bar = 50 *μ*m); (b) The pathological score in each group of mice was quantified; (c) The wet weight/dry weight ratio of skeletal muscle was calculated to assess the extent of edema in different groups of mice. (d) The level of LDH was measured in muscular homogenates of each group of mice; (e) The level of CK-MB was measured in muscular homogenates of each group of mice. *n* = 10 for each group. ^*∗*^*p* < 0.05 and ^*∗∗*^*p* < 0.01.

H&E staining data showed intact borders regularly arrayed without breaks, holes, and edema, which identified healthy fibers. Injured fibers were confirmed by edema and broken and fragmented fibers. I/R mice showed muscle fiber degeneration, dissolution, sarcoplasm, and inflammatory cell infiltration along with myoedema; whereas, healthy fibers were observed in the mice of the sham group. Meanwhile, NBP treatment reduced the inflammation degree of muscular tissue (Figure 1(a)). Consequently, NBP treatment alleviated the I/R-induced pathological alterations in muscular tissues (Figure 1(b), *p* < 0.05).

Furthermore, mice in the I/R group presented with a higher W/D ratio for the skeletal muscle tissue compared to mice in the sham group (Figure 1(c), *p* < 0.05). In contrast, the W/D ratio was lower in the NBP group than that in the I/R group (Figure 1(c), *p* < 0.05).

Moreover, elevated levels of LDH and CK-MB were found in the I/R group versus the sham group, suggestive of muscle damages along with pathological alterations following I/R injury. On the other hand, NBP treatment led to contrasting results (Figures 1(d) and 1(e), *p* < 0.05).

### 3.2. NBP Ameliorated Skeletal Muscle Inflammatory Response in Mice with Limb I/R Injury

**Figure 2:**
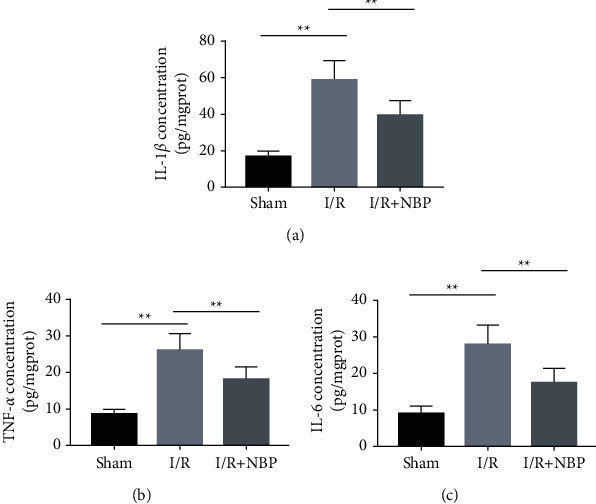
Effect of NBP on inflammatory responses of skeletal muscles in I/R-injured mice. (a) (b) and (c) ELISA was conducted to determine the levels of IL-1*β* (a), TNF-*α* (b), and IL-6 (c) in the homogenates of muscular tissues of each group of mice; *n* = 10 for each group. ^*∗*^*p* < 0.05 and ^*∗∗*^*p* < 0.01.

Additional assessment revealed higher levels of IL-1*β*, TNF-*α*, and IL-6 in the I/R group relative to those in the sham group. Conversely, NBP therapy reversed the increased inflammatory cytokine levels (Figure 2, *p* < 0.05).

### 3.3. NBP Alleviated Oxidative Stress in the Skeletal Muscles of Mice with Limb I/R Injury

**Figure 3:**
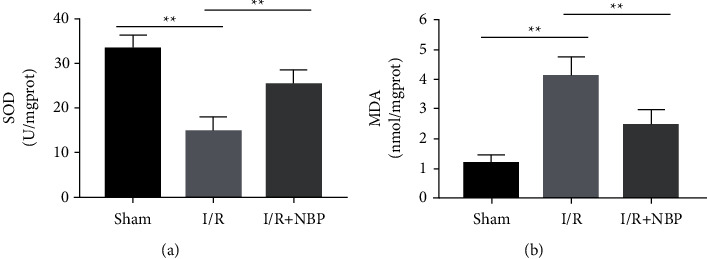
Effect of NBP on oxidative stress of skeletal muscles in I/R-injured mice. (a) The production of MDA was measured in muscular homogenates of each group of mice; (b) The activity of SOD was measured in muscular homogenates of each group of mice. *n* = 10 for each group. ^*∗*^*p* < 0.05 and ^*∗∗*^*p* < 0.01.

Relative to the sham group, MDA production was increased in I/R group but it was lowered in the NBP group (Figure 3(a), *p* < 0.05). Moreover, lower SOD activity was observed in the I/R group when compared to those in the sham group. Meanwhile, the NBP group had higher SOD activity than the I/R group (Figure 3(b), *p* < 0.05).

### 3.4. NBP Inactivated the HMGB1/TLR4/NF-*κ*B Pathway in Mice with Limb I/R Injury

**Figure 4:**
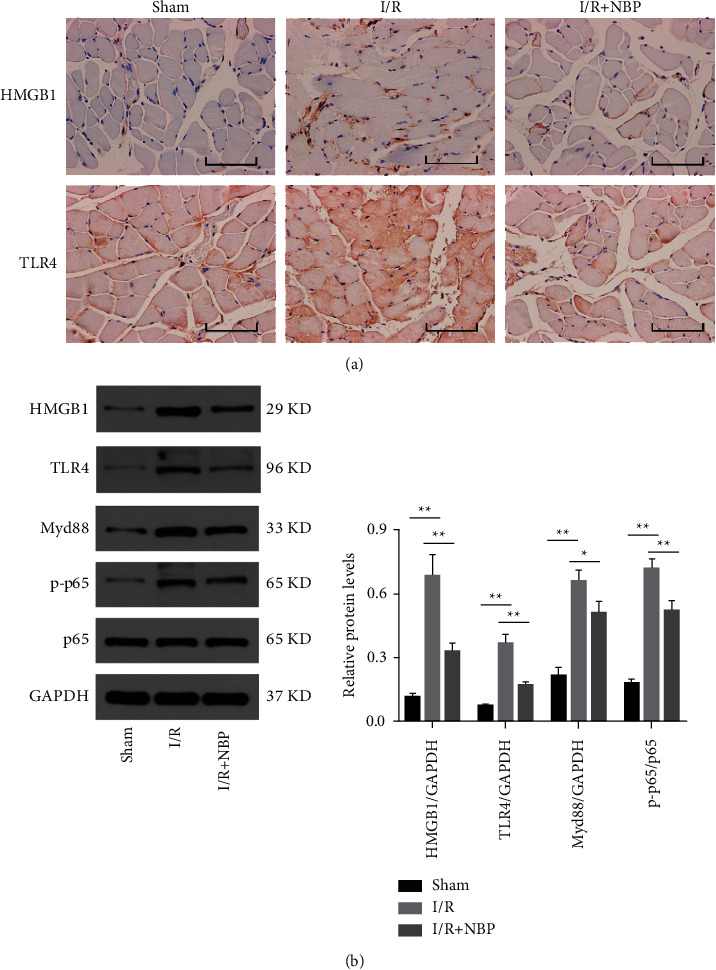
Effect of NBP on activation of the HMGB1/TLR4/NF-*κ*B pathway in I/R-injured mice. (a) Immunohistochemistry was conducted to assess the quantity of HMGB1 and TLR4 positive cells in each group of mice (bar = 50 *μ*m); (b) Immunoblotting was used to examine the protein expressions of HMGB1, TLR4, Myd88, and the extent of NF-*κ*B p65 phosphorylation in the muscular homogenates in each group of mice. *n* = 3 ^*∗*^*p* < 0.05and ^*∗∗*^*p* < 0.01.

Furthermore, the impact of NBP treatment on HMGB1/TLR4/NF-*κ*B in the muscular tissues of different groups of mice was investigated 72 h after treatment with immunohistochemistry and immunoblotting (Figure 4(a) and 4(b)).

While lower HMGB1 and TLR4 levels were expressed in the sham group, elevated HMGB1 and TLR4 levels were documented in the I/R group. Additionally, in contrast to the I/R group, the quantity of HMGB1 and TLR4 positive cells was diminished in the NBP group (Figure 4(a)).

As illustrated in Figure 4(b), the I/R group had elevated protein expressions of HMGB1, TLR4, Myd88, and extent of p65 phosphorylation compared to the sham group. However, these elevations were abolished in the NBP group relative to the I/R group, indicating that NBP treatment, for the most part, repressed the HMGB1/TLR4/NF-*κ*B pathway activation in the muscular homogenate, adding to its defensive influence against limb I/R injury.

## 4. Discussion

**Figure 5:**
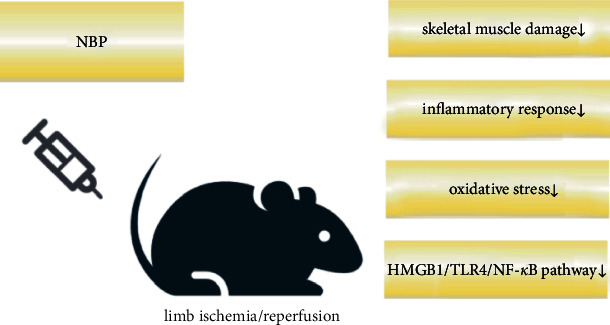
In summary, this study dissected out the effect of NBP on the limb I/R injury-driven skeletal muscle damages, and our findings suggested that NBP could protect limbs against I/R injury by inhibiting inflammation responses and oxidative stress *via* the HMGB1/TLR4/NF-*κ*B pathway.

Limb I/R injury-induced oxidative stress, which is accompanied by inflammatory response, contributes to profound muscular tissue dysfunction. The current study was performed with the goal to explore whether NBP lessened inflammation induced by limb I/R injury and mitigated tissue edema in skeletal muscles. Accordingly, our findings indicated that NBP ameliorated inflammatory responses and oxidative stress was induced by limb I/R injury. Moreover, we uncovered mechanistically that NBP inactivated HMGB1/TLR4/NF-*κ*B in the skeletal muscle of limb I/R injury, highlighting that protective effects of NBP were attributed to repression of HMGB1/TLR4/NF-*κ*B (Figure 5).

Limb I/R injury-driven skeletal muscle damage is a clinical challenge that requires much attention [1–3]. Currently, several approaches are employed to treat limb I/R injury, which include the use of physical and chemical treatment regimens. In addition, hypothermia, ischemic preconditioning, ischemic postconditioning, controlled reperfusion, and light-emitting diode therapy possess the ability to alleviate limb I/R injury-driven skeletal damage [18–20]. Some medications, such as curcumin, dexamethasone, simvastatin, silibinin, cyclosporine A, and saline [21–23], are also known to be effective to reduce limb I/R injury-driven skeletal damage. The usage of such methods remains limited in the horrendous wound. In specific cases, especially in severe extremity injuries, surgery is warranted to prevent mortality hemorrhage to protect the function of major organs. More importantly, while the above mentioned procedures and pharmaceutical regimens have been demonstrated to be successful during research, none have been identified effective in the clinical settings. In lieu of the same, a pressing need exists to dissect out novel agents with anti-inflammatory and antioxidant characteristics that can be adopted for the treatment I/R injury-driven skeletal muscle damage.

NBP is known to be clinically effective against ischemic stroke. The adoption of NBP in general clinical use is attributed to its broad range of characteristics, including the features of the antioxidation [14], antiapoptosis [15], and the ability to mitigate inflammation [16]. Of note, there are many evidences to suggest that NBP could mitigate brain edema induced by concussive head injury [24]. In addition, prior data have further demonstrated that NBP can guard against cerebral I/R injury-triggered edema development by breaking down the blood-brain barrier [25]. Herein, our findings unveiled that NBP alleviated the limb I/R injury-driven damage of skeletal muscle tissues in mice. Additional histological assessment illustrated fewer pathological changes in the presence of NBP. Moreover, our findings revealed that NBP could alleviate edema in the skeletal muscle of limb I/R injury.

Furthermore, inflammatory responses are essential for the pathogenesis of skeletal muscle I/R injury characterized by infiltration of inflammatory cells [4]. Similarly, penetrating neutrophils deliver an assortment of proinflammatory cytokines; for instance, IL-1*β*, TNF-*α*, and IL-6 are capable of aggravating inflammatory responses [26]. In addition, numerous studies have indicated the ability of NBP to alleviate inflammation in cerebral I/R injury and other illnesses associated with inflammatory responses [27, 28]. Much in accordance with the same, our findings illustrated that NBP could mitigate the degree of inflammatory reactions in the skeletal muscle tissues caused by limb I/R injury.

Oxidative stress, a consequence of imbalance between production and accumulation of ROS in cells, is remarkably imperative in the advancement of the limb I/R injury process [6]. Experimentation in our study indicated that NBP could significantly diminish oxidative stress of skeletal muscle triggered by limb I/R injury by lessening MDA production and augmenting SOD activity. In line with the current discovery, a prior study documented the antioxidative properties of NBP in the brain of patients with Alzheimer's disease [29]. Moreover, NBP was previously shown to relieve anxiety and depression-like behaviors through the restriction of oxidative stress [30]. All the aforementioned studies are indicative of the usage of NBP in the management of limb I/R injury-driven skeletal muscle damage due to its antioxidative properties.

Activation of the HMGB1/TLR4/NF-*κ*B pathway has been previously observed under inflammatory responses or oxidative stress conditions [31]. Besides, the HMGB1/TLR4/NF-*κ*B pathway is likewise activated in various I/R models [32]. Herein, our findings demonstrated increased protein expressions of HMGB1, TLR4, Myd88, and extent of p65 phosphorylation in the I/R group, whereas the declines were documented in the NBP group. The efforts of Zhang et al. revealed that NBP treatment essentially enhanced cerebral I/R-triggered brain injury by restraining TLR4/NF-*κ*B-related inflammatory responses [33]. Additionally, He et al. showed that NBP decreased activation of BV2 cells, reduced the release of inflammatory cytokines, and further restrained the expression of TLR4/NF-*κ*B in BV2 cells, consequently safeguarding against spinal cord injury. Nonetheless, none of these studies focused on the effects of NBP on inflammatory responses through HMGB1. Accordingly, our study is the first-of-its-kind to reveal that NBP treatment inhibited the protein expression of HMGB1, resulting in repressing I/R-induced muscular injury.

## 5. Conclusions

In summary, this study dissected out the effect of NBP on the limb I/R injury-driven skeletal muscle damages, and our findings suggested that NBP could protect the limbs against I/R injury by inhibiting inflammation responses and oxidative stress *via* the HMGB1/TLR4/NF-*κ*B pathway. Additionally, our discoveries highlight the potential of NBP to serve as an effective strategy against I/R injury-driven skeletal muscle tissue damages.

## Data Availability

All data generated or analyzed during this study are available from the corresponding author upon reasonable request.
